# Implementation of a complex eHealth intervention by a public–private partnership in clinical practice: a qualitative multicentre analysis using CFIR

**DOI:** 10.1093/her/cyaf001

**Published:** 2025-02-21

**Authors:** R V H IJzerman, R van der Vaart, L D Breeman, R A Kraaijenhagen, A W M Evers, W J M Scholte op Reimer, V R Janssen

**Affiliations:** Leiden University, Institute of Psychology, Health, Medical and Neuropsychology Unit, Wassenaarseweg 52, Leiden 2333 AK, The Netherlands; Department of Cardiology, Amsterdam UMC, University of Amsterdam, Meibergdreef 9, Amsterdam 1105 AZ, The Netherlands; Leiden University, Institute of Psychology, Health, Medical and Neuropsychology Unit, Wassenaarseweg 52, Leiden 2333 AK, The Netherlands; The Hague University of Applied Sciences, Centre of Expertise Health Innovation, Johanna Westerdijkplein 75, Den Haag 2521 EN, The Netherlands; Leiden University, Institute of Psychology, Health, Medical and Neuropsychology Unit, Wassenaarseweg 52, Leiden 2333 AK, The Netherlands; NDDO Institute for Prevention and Early Diagnostics (NIPED), Teleportboulevard 140, Amsterdam 1043 BZ, The Netherlands; Vital10, Courbetstraat 34, Amsterdam 1077 ZV, The Netherlands; Leiden University, Institute of Psychology, Health, Medical and Neuropsychology Unit, Wassenaarseweg 52, Leiden 2333 AK, The Netherlands; Medical Delta, Leiden University, Technical University Delft, Erasmus University Rotterdam, Huismansingel 4, Delft 2629 JH, The Netherlands; Department of Cardiology, Amsterdam UMC, University of Amsterdam, Meibergdreef 9, Amsterdam 1105 AZ, The Netherlands; HU University of Applied Sciences, Research Group Chronic Diseases, Heidelberglaan 7, Utrecht 3584 CH, The Netherlands; Leiden University Medical Centre, Department of Cardiology, Albinusdreef 2, Leiden 2333 ZA, The Netherlands

## Abstract

Complex eHealth interventions—featuring multiple components within dynamic systems—are used for healthcare improvement. Public–private partnerships (PPPs), combining resources, expertise, and technology, are crucial in this context. Yet, integrating these interventions into practice remains challenging. This study identifies barriers and facilitators affecting implementation of the BENEFIT programme, a complex eHealth intervention targeting cardiovascular disease patients, by PPP within practice. A qualitative study design was employed. Ten key stakeholders from four cardiac rehabilitation (CR) sites, who were all PPP partners involved in developing and implementing the BENEFIT programme, were interviewed semistructured. Transcripts were analysed using Consolidated Framework for Implementation Research. Facilitators included programme adaptability, communication and planning within teams, digital healthcare needs, dedicated PPP leadership, PPP meeting structure and PPP’s ability to quickly modify the implementation strategy. Barriers involved specific PPP challenges (frequently changing roles, unclear roles and responsibilities and limited staffing), workplace disruptions, poor information technology (IT) integration, and ambiguous implementation goals amongst CR sites. This case study highlights challenges in implementing complex eHealth interventions by PPPs within practice. The findings underscore the need for a comprehensive implementation approach considering specific PPP dynamics, including combined expertise and resources, transparent role definition, sufficient staffing, clear goal communication and adaptable strategies for sustainable implementation.

## Introduction

Complex eHealth interventions are promising in promoting healthy lifestyle behaviours to improve individuals’ health and quality of life and alleviating the healthcare system [1]. Integrating complex eHealth interventions in routine practice, however, presents a multifaceted challenge since implementing complex eHealth interventions is inherently difficult [2–4]. One primary contributor is the eHealth interventions complex nature [1] based on different elements, such as the kind of health behaviours it aims to modify, the groups or populations it aims to reach, the extent of intervention adaptability and the professional competence and skills needed to implement the intervention [5]. Thereby, complexity is based on the dynamics between the intervention and its surrounding environment. This encompasses all aspects in which an intervention is conceptualised, created, implemented and evaluated. Furthermore, implementing complex eHealth interventions is challenging due to the multiple interactions of behavioural, technological and organisational intervention components [6], which must be coordinated at various levels to work effectively [1, 6, 7]. Frequently identified challenges are poor planning, cost of (implementing) the intervention, organisational misalignment and ineffective collaboration between key stakeholders [2, 3, 7, 8].

To unlock complex eHealth interventions’ full potential, a collaborative approach is necessary. Public–private partnerships (PPPs) have emerged as a vital approach to improving healthcare services worldwide [9–11]. PPPs often involve public authorities, e.g. governments or government organisations such as universities or academic medical centres, researching to systematically enhance and evaluate innovations, and the private sector developing innovative healthcare solutions, infrastructure or other assets critical for delivering public services [10], amongst others, focused on treatment or prevention of chronic illnesses such as cardiovascular disease (CVD) [12, 13]. Successful PPPs provide infrastructure, funding, expertise and technology [9, 14]. They contribute to increased healthcare system efficiency and access, decreased costs and enhanced innovation [9]. Research has shown that PPPs are instrumental in addressing resource shortages by improving public health systems’ quality, provision and performance of health services [11, 14]. This helps integrate public and private sector facilities and strengthen health systems [11]. However, despite their potential, PPPs require specialised skills such as effective governance and strategies [14]. Effective organisation and government support are essential for successful PPPs, yet ambiguities surrounding the private sector’s role and the extent of its benefits in these alliances often impede stakeholder confidence. This is particularly true amongst healthcare policymakers, who are still hesitant to initiate such partnerships [11]. Moreover, initial financial assessments rarely account for unforeseen complexities that are specific to PPP collaborations, which can lead to budgetary overruns and extended timelines [15, 16]. Evidence has shown a tendency for future revenues to be excessively committed, such as entering contracts that are too expensive for government bodies, leading to the costs being passed on to the consumers [13]. Nevertheless, as public health budgets are increasingly under pressure, the World Health Organization recommends using PPPs [13]. Therefore, the need for comprehensive research into the dynamics of a PPP when implementing complex eHealth interventions becomes even more pressing. This knowledge stimulates effective and efficient collaboration within PPPs and supports implementing complex eHealth interventions into existing healthcare systems, thereby optimising patient outcomes and maximising the benefits of such interventions [17].

Implementation science can be used to systematically evaluate challenges of implementing complex eHealth interventions by PPPs within clinical practice [18]. By applying the Consolidated Framework for Implementation Research (CFIR) [19], we can systematically analyse the nuanced layers of PPP operations and explore multifaceted factors that influence the implementation of complex eHealth interventions, encompassing both barriers and facilitators inherent in the process. Therefore, this study uses CFIR as a guiding framework to delve into the implementation process of a complex eHealth intervention by a Dutch PPP, aiming to enhance our understanding of PPP dynamics to optimise future implementations of complex eHealth interventions into clinical practice.

## Methods

### Case study

The BENEFIT for all intervention programme, referred to as the BENEFIT programme, is developed by a PPP as an addition to cardiac rehabilitation (CR) and aims to promote and support sustainable healthy living at home amongst individuals with CVD in the Netherlands [20, 21]. This PPP brings together contributions from academia, a private party and healthcare: academia provided evidence-based input for the BENEFIT programme and the associated eHealth platform, the private party developed a Personal Health Application (PHA) and provided a reward ecosystem and the digital infrastructure essential for implementing the intervention, and healthcare facilitated the implementation and use of the intervention within routine clinical practice, specifically, CR.

Central to the BENEFIT programme is the PHA, which offers various features that are part of an ecosystem and link different necessary pillars to facilitate sustained healthy living. This includes access to evidence-based lifestyle interventions, daily goal monitoring, coaching, and a reward program designed to incentivise health behaviours and adherence. This way, healthy living is made more appealing by introducing challenges and rewards for, amongst others, attending healthcare appointments and monitoring lifestyle behaviours. Rewards can be redeemed for discounts on health-related goods and services. Given the different pillars and options, the PHA can be tailored to the specific needs and preferences of end users. In the context of CR, the BENEFIT programme is implemented as a complementary component to standard care. The intervention enables healthcare providers to offer patients information, feedback, remote healthcare services and assistance in selecting suitable lifestyle interventions. Patients could continue using the BENEFIT programme after their CR was completed to facilitate long-term maintenance of new health behaviour changes. This aims to permanently perpetuate newly learned healthy lifestyle behaviours [20, 21].

Due to the coronavirus disease 2019 (COVID-19) outbreak in March 2020, a rapid transition to digital healthcare delivery was necessary, and telerehabilitation was provided using the PHA. Standard CR care resumed in February 2021, integrating in-person care and digital care using the BENEFIT intervention programme.

### Study population and design of the current study

A formative evaluation [22] was conducted to identify barriers and facilitators affecting the implementation of the BENEFIT programme developed by a PPP in clinical practice. The purposeful sample consisted of 10 stakeholders (researchers, company board members and healthcare providers) from four CR sites, representing key roles during the implementation process, i.e. they formed the core implementation team in various CR sites and consisted of both the public and private sector of the PPP, ensuring that essential insights from academic, business and healthcare perspectives were represented. The sample included a researcher/coordinator (academia), a researcher/clinical psychologist (academia), a CR director/medical specialist (business), two implementation managers (business), a site manager (healthcare), specialised nurse/coordinator of patient care (healthcare), a hospital department head/medical specialist (healthcare), a physiotherapist (healthcare) and a specialised nurse (healthcare).

When implementation tasks of the BENEFIT programme were being completed, invitations were sent via e-mail along with an information letter (description of the purpose of the interview, the use of the data and the duration of the interview) and the requirement for online informed consent using Qualtrics, and in-depth understanding of the implementation process from their perspective was gained. Using a qualitative study design, between May 2022 and September 2022, one-on-one semistructured interviews were executed with the participants. This provided flexibility in our questioning and enabled thorough exploration of topics based on the five implementation domains of the CFIR [6–8]: (i) Innovation: the intervention that is implemented, such as design features, perceived advantages and potential adaptations within clinical practice; (ii) Outer setting: the environments in which the inner setting operates, such as a hospital system, healthcare policies, economic forces and patient needs; (iii) Inner setting: the organisation adopting the intervention, e.g. hospital unit(s), and its culture, structural characteristics and intervention readiness; (iv) Individuals: the individuals involved, considering their roles, interests they represent, beliefs and behaviours when interacting with the intervention; and (v) Implementation process: activities and strategies used by the PPP to implement the innovation, including detailed planning, assessing context, execution and ongoing evaluation. This approach enabled the systematic examination of facilitators and barriers that impacted the implementation process in the different healthcare sites [19]. Building on CFIR research [23, 24], primary author R.V.H.IJ., a female researcher with a background in psychology and trained in questioning and interviewing, developed the interview protocol (Appendix 1), which was subsequently improved in collaboration with R.v.d.V., V.R.J., and W.J.M.S.o.R. Our semistructured interview approach was designed to balance comprehensive coverage of all CFIR domains whilst maintaining participant engagement. We began with open-ended questions to allow participants to reflect on their experiences freely, followed by tailored follow-up prompts to ensure that all CFIR constructs were discussed if relevant. For each domain, a detailed list of all CFIR constructs was visible to participants on paper or screen, facilitating a structured yet conversational discussion after we first reviewed them together. Interviews were performed in Dutch by R.V.H.IJ All interviews took place face-to-face at the healthcare site or via Microsoft Teams. Interviews lasted ∼60–90 min, were audio recorded and were transcribed verbatim afterwards.

### Content of the interviews

Before the start of the interview, R.V.H.IJ. discussed the purpose of the study and the interview setup. Subsequently, the interview consisted of two parts. The first part consisted of an examination of the participant’s general role within the PPP and the tasks involved, their assigned implementation role(s) and the tasks involved, possible implementation tasks outside their assigned role(s), their vision of the main reason of implementing the BENEFIT programme within their organisation and their perspective on conditions needed to implement the BENEFIT programme successfully. The second part consisted of questions based on the five CFIR domains [19] to systematically identify facilitators and barriers of the PPP and the implementation of the BENEFIT programme. Discussing each domain separately, participants reflected on their experiences with the implementation successes and challenges. Follow-up questions were asked to ensure that all subjects were discussed.

### Data analysis

**Fig. A1.**
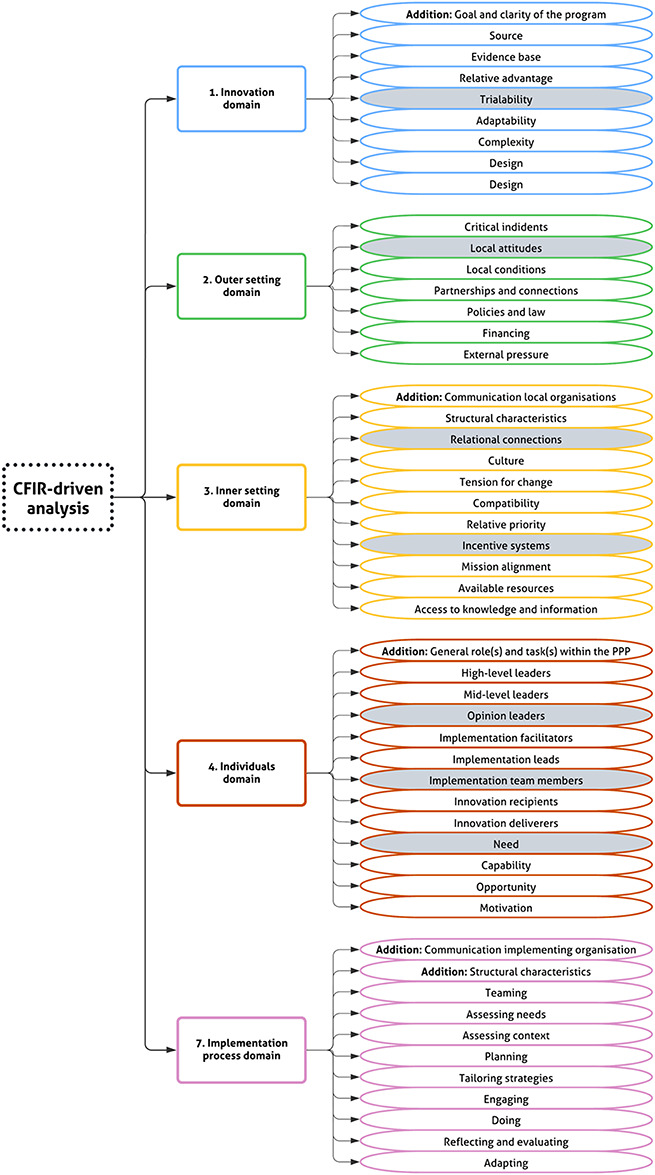
Coding tree. CFIR-driven analysis. The boxes containing a white background represent all codes that emerged during the analysis. All boxes containing a dark background did not emerge during the analysis.

Data analysis was conducted using MAXQDA 2023 [25]. The template analysis method is a variant of thematic analysis, which underscores hierarchical coding and offers a structured approach to textual data analysis whilst providing the adaptability required for specific research requirements, which was used to interpret the data [26]. Initially, the 48 CFIR constructs and subconstructs were used as preliminary codes in our coding manual. Five additional codes were derived from the data: (i) Goals and clarity of the program (added to Innovation), (ii) Communication local organisation (added to the Inner setting), (iii) PPP role(s) and task(s) (added to Individuals), (iv) Communication implementing organisation (added to the Implementation process) and (v) Structural characteristics (added to the Implementation process). The codes added to Innovation, Inner setting and Individuals were integrated based on their emerging significance in clarifying influential factors at different levels within the implementation context. For clarity during the coding process, we opted to categorise influencing factors related to the PPP under the domain Implementation process and influencing factors related to the cardiac sites where the implementation occurred under the domain Inner setting. Since two constructs of the Inner setting also proved to be relevant to the PPP during data analysis, these were, therefore, also incorporated into the domain Implementation process. Appendix 2, Fig. A1. outlines the coding structure.

Interview analysis proceeded in three steps. First, R.V.H.IJ. and V.R.J. independently categorised interview fragments to CFIR constructs. Every discrepancy in the choice of construct and domain was discussed until consensus was reached. Summaries for each construct were then independently done by R.V.H.IJ. and R.v.d.V. for the first constructs. Then, any discrepancies in summaries for each construct were discussed until consensus was reached. Finally, summaries of all constructs from each domain were merged. Influencing factors for each domain were categorised as either facilitators or barriers. Guidelines provided by the Consolidated Criteria for Reporting Qualitative Research checklist (Appendix 3) were used as guidance to ensure quality and accuracy of the findings [27].

## Results

### Participants’ characteristics

Of the 10 participants, eight were female and two were male. Most participants played dual PPP roles, which added a multidimensional perspective to the study. Participants’ tasks included clinical care, patient counselling, implementing new systems, programme coordination, involvement in research and healthcare innovation and financial and management functions. The sample represented a wide range of implementation roles, as defined by CFIR, and tasks that contributed to implementing the BENEFIT programme. High-level leader participants had programme coordination responsibilities, project management involvement, substantive contributions regarding development and implementation of the programme and decision-making responsibilities. Mid-level leaders monitored, coordinated and facilitated implementation with attention to problem identification, communication and healthcare provider support. Implementation leads were responsible for system design, coordination and adjustments, as well as successful programme implementation. Implementation facilitators streamlined workflows, provided user training and customised the used systems. Innovation deliverers helped evaluate, guide and adapt processes to implement the BENEFIT programme more effectively.

### Influencing factors

To provide a thorough and systematic understanding of the factors that impacted the PPP and the implementation of the BENEFIT programme, each CFIR domain is described separately. Tables outline influencing factors for each domain. Factors that exerted the most notable influence on the implementation process or were frequently cited by participants are termed ‘key barriers’ and ‘key facilitators’. Other factors identified (Appendix 4) as having an impact are simply referred to as ‘barriers’ and ‘facilitators’.

#### CFIR Domain 1: Innovation

This section outlines participants’ views on the influence of the domain Innovation: the BENEFIT programme, developed and implemented by the PPP within clinical practice. Table I describes all key factors that proved to be relevant during the implementation, along with exemplary quotes from participants illustrating these factors.

**Table I.** Key factors within CFIR Domain 1: Innovation influencing implementation

Theme	Quotes
**Key facilitator**	
Programme adaptability	‘We have been remarkably flexible with the programme; we were able to highly adapt and fine-tune it to align with the environmental context as well as the patients’ needs.’
**Key barrier**	
Lack of clarity and uniformity about the main reason for implementing the programme within organisations	‘The most significant obstacle has been the ambiguity surrounding the exact nature of the BENEFIT programme. This was not clear.’ ‘We were very much supported in the implementation and use of digital healthcare, predicated on the assumption of cost savings and space efficiency. However, without framing it as a quality enhancement, a new improvement will not succeed. Initially, it always demands more time, resources, and financial investment.’

A key facilitator was programme adaptability; the programme proved to be capable of being tailored to various environments, situations and patient needs. This was key in ensuring the relevance of the intervention across different contexts.

A key barrier was the lack of clarity and uniformity regarding the main reason for programme implementation in different organisations. Some thought that it was implemented as an alternative to regular healthcare, whilst others considered it an additional resource on top of their routine cardiac care or mentioned that its goal was to reduce healthcare costs and physical space requirements in regular cardiac care. The board’s emphasis on cost reduction created misalignment with healthcare providers’ principal aim of improving patient care, leading to confusion about the programme’s purpose within the organisation. Furthermore, within the PPP, determining the most optimal programme role within standard care (such as whether it should be optional or standard part of routine care) was a protracted effort which possibly affected clear communication of the programme’s main objective to external partners.

#### CFIR Domain 2: Outer setting

This section outlines participants’ views on the influence of the domain Outer setting: the environment in which the inner setting operates. Table II presents all identified key factors, along with exemplary quotes.

**Table II.** Key factors within CFIR Domain 2: Outer setting influencing implementation

Theme	Quotes
**Key facilitator**	
The need for digital healthcare	‘When direct contact in cardiac rehabilitation was no longer possible, the availability of an online programme was exceptionally fortunate. So, the COVID-19 pandemic served as a good incentive.’ ‘In this specific instance, it was the Dutch Heart Foundation that was particularly instrumental in promoting the programme, as they were very pleased with the availability of this option. This has certainly yielded benefits.’
**Key barrier**	
Workplace disruptions	There was a ‘considerable flux. Not solely within the BENEFIT programme, but the healthcare landscape and its personnel had also undergone a huge transformation. Such extensive change is overwhelming.’ ‘Initially, we compared a supplement/control condition with a face-to-face and online intervention. However, the advent of COVID-19 necessitated a large-scale shift to telerehabilitation. This turnaround in implementation had significant consequences. An organisation that is naturally resistant to rapid change needs time to build consensus. Changing even one protocol meets resistance; staff need to be retrained and reoriented to new procedures. Therefore, try to keep changes to a minimum.’ ‘We were compelled to revert to the control condition, a process that required considerable effort. We devised a strategic plan addressing the involved parties: who are they, what resources do they require to facilitate this transition, and what is the underlying justification? Within the PPP, this led to a negotiation, balancing the ideal with the practical, and finding a middle ground where both could meet. Ultimately, we succeeded in incorporating a control group in such a manner that it was acceptable to the organisations involved.’

The COVID-19 pandemic had a dual impact. A key facilitator was the ‘acute need for digital healthcare’ due to sudden unavailability of face-to-face contact caused by the COVID-19 pandemic. The private party was well positioned compared to most hospitals in the Netherlands in this regard. Therefore, thanks to the PPP, it was possible to quickly scale up the digital healthcare programme. As a result, the Dutch Heart Foundation recommended using the BENEFIT programme at a national level. However, the pandemic also introduced significant ‘workplace disruptions’, which were identified as a key barrier. The healthcare environment experienced drastic changes, requiring staff to suddenly adapt to new working methods. Combined with the use of the BENEFIT programme in a different way than initially planned, all changes and obligations were experienced as a huge challenge. The main underlying reasons for these challenges were the conflicting PPP interests; the academic partner wanted a scientific evaluation, and therefore, the implementation of a baseline measurement was required. Current healthcare circumstances dictated otherwise, given the need for digital healthcare. This presented logistical challenges which were resolved by first switching to digital healthcare, and later, after COVID-19 restrictions, switching back and still conducting the baseline measurement. Extra time and effort to bring everyone on board with these new methods were needed.

#### CFIR Domain 3: Inner setting

This section outlines participants’ views on the influence of the domain Inner setting: the organisations where the BENEFIT programme was implemented. Table III describes all identified key factors, along with exemplary quotes.

**Table III.** Key factors within CFIR Domain 3: Inner setting influencing implementation

Theme	Quotes
**Key facilitator**	
Communication and planning in local team	‘We convened at regular intervals, engaging in reflective practice and evaluation. Meticulous planning was evident, with all tasks meticulously catalogued on the Trello board, affording our team leader a comprehensive overview.’
**Key barrier**	
Poor information technology (IT) integration	‘Using two systems was not conducive to a successful implementation, it was rather counterproductive.’ ‘It led to duplicate filing, which emerged as a formidable challenge. The labour and time involved were unsustainable, to be candid.’

A key facilitator within certain organisations was effective communication within the local team. They organised regular meetings, reflections and evaluations to ensure alignment and progress. Using tools such as Trello for planning and oversight was also mentioned as instrumental in keeping the team organised and on track.

A key barrier found was poor information technology (IT) integration; participants reported significant challenges due to duplicate filing and the need to work across multiple systems, which led to increased workload and inefficiencies.

#### CFIR Domain 4: Individuals

This section outlines participants’ views on the influence of the domain Individuals: all roles and characteristics. Table IV describes all identified factors, along with exemplary quotes.

**Table IV.** Key factors within CFIR Domain 4: Individuals influencing implementation

Theme	Quotes
**Key facilitator**	
Dedicated PPP leaders with multiple roles	‘As a [B1] and the founder and director (...), my primary focus is on medical oversight, fostering collaboration, and the development of our programme, ensuring that I remain actively engaged with the content. (…) In addition, I continue to practice clinically. I also serve as the lead cardiologist at our cardiac rehabilitation sites. This role involves being accountable for the financial health of our organisation, which includes obligations to both health insurers and shareholders. Regarding the BENEFIT programme, as project leader, I am a member of the steering committee. My position as the lead [B1] for our cardiac rehabilitation sites also entrusts me with the final decision-making authority regarding the programme’s implementation.’ ‘As a [B14] and [B19] within the (...), my role encompasses patient care, leading various hospital projects, active involvement in numerous healthcare innovation initiatives, and overseeing the academic progress of several PhD students. In relation to the BENEFIT project, I hold the position of one of the project leaders, so I am part of the steering committee. My responsibilities in this capacity include steering the project towards a successful finish. This involves various tasks, such as being accountable for the research design, managing both the project and the broader research programme, contributing to the execution and supervision of research activities, and overseeing the communication strategy. I have also played a significant role in the implementation process of the BENEFIT programme.’
**Key barrier**	
Changing roles within the PPP	‘Due to our team’s size, we frequently had to alter roles. We are fulfilling more than a fixed set of tasks. Recently, I assumed the position of [B13].’ ‘The most challenging aspect, I believe, pertains to [L0]’s domain. Occasionally, I was drawn into situations where the programme faltered, and I was asked to intervene without knowledge and context, yet expectations remained.’

A key facilitator was the presence of dedicated PPP leaders with multiple roles. For example, on the one hand, they were involved in programme development, but at the same time, closely involved in programme implementation because they oversaw implementation sites. This PPP construction brought great advantage because individuals were involved from different PPP perspectives, and coordination of internal and external matters could occur within the steering committee itself.

A key barrier identified was the need for individuals to frequently switch roles. This was prevalent within smaller organisations and within the PPP. Individuals were often required to adapt to different roles, sometimes abruptly and without adequate preparation or understanding of the underlying issues. This put pressure on them to perform tasks for which they might not have felt fully equipped.

#### CFIR Domain 5: Implementation process

This section outlines participants’ views on the influence of the domain Implementation process: the activities and strategies used to implement the innovation. Table V describes all identified factors, along with exemplary quotes.

**Table V.** Key factors within CFIR Domain 5: Implementation process influencing implementation

Theme	Quotes
**Key facilitators**	
A diligent PPP meeting structure	‘Our schedule included monthly evening sessions dedicated to comprehensive reviews, alongside weekly gatherings, and bilateral meetings between research leads. Furthermore, meetings between research leaders and staff were a regular occurrence, facilitating frequent consultations at all levels. Additionally, we maintained consistent communication with the stakeholder committee, its chairperson, and naturally, with the patient panel and healthcare providers at various sites.’
The PPP adaptability regarding the programme’s implementation strategy	‘With people needing healthcare but unable or reluctant to visit facilities in person, remote care became essential. Fortuitously, the environment provided by the BENEFIT programme was well-prepared, prompting us to deploy the intervention immediately. To have persisted with the control condition, as initially planned, would have been unethical.’
**Key barriers**	
A newly established PPP	‘In the context of a public-private partnership, which is in line with establishing a whole new organisation, substantial investment is required. We must deliberate on collaborative methods, communication channels, and decision-making processes, and ensure their execution. This essentially means constructing a new organisation from the ground up, a vital learning curve for us.’ ‘Transparent communication about objectives and interests is critical, though simultaneously challenging due to the inherent political dynamics. I am convinced that focussing on open dialogue and aligning on shared goals will lead to greater clarity, efficiency, and satisfaction, ultimately conserving resources and reducing frustration.’ ‘The engagement of an organisational consultant from the outset would have been advantageous, to guide us through the complexities of such collaboration, which we were navigating together.’
Unclear roles and responsibilities within the PPP	P3: ‘I had no specific tasks related to implementation. Other than my sole duty being to inform those who would utilise the BENEFIT programme.’ *This contrasts with others*: P2: ‘[P3] was chiefly responsible for the implementation across various sites.’ P4: ‘Indeed, [P3] was instrumental in the implementation alongside [P2]. They visited the sites to conduct training sessions. From what I gather, these efforts were successful, but beyond that, my knowledge is limited.’ R.V.H.IJ.: ‘Had there been a premeditated plan detailing individual responsibilities for the implementation tasks and their execution?’ P2: ‘There was a strategy in place, though in retrospect, it warranted further refinement. We got [P3] involved from the outset, and my involvement was equally deliberate and considered.’
Limited staffing capacity within the PPP to implement the programme	‘Of course, there was also a lot still going on, on the outside, necessitating adaptations due to staff turnover and dynamic circumstances.’ ‘Effective implementing and executing hinge on the availability of a competent team with the necessary time allocated. It is imperative that projects must be budgeted and staffed to facilitate feasible execution.’ ‘It was an oversight on our part, as a public-private partnership, not to have predetermined the budget allocation for the implementation itself. Clarifying the financial and personnel contribution from the private party, as well as the role of researchers, would have been prudent. A more transparent division of responsibilities and resources between research funding and private contributions was needed.’

A key facilitator was the diligent meeting structure within the PPP, which included monthly review sessions, weekly meetings and regular bilateral discussions amongst PPP leaders. This ensured that all three parties and perspectives were consistently considered during implementation; the programme was scientifically evaluated, the perspectives of healthcare and patient interests were considered and the private party focused on creating sustainable funding for the programme and tapping into relevant contacts within the ecosystem. For example, there were weekly consistent meetings on Tuesday mornings involving research leaders and staff members, ensuring ongoing dialogue at multiple organisational levels. Since project teams worked across disciplines, implementation ideas could be tested scientifically and then implemented within practice quickly. Also, in case of challenges, they could collaboratively devise solutions and swiftly implement necessary actions. This ensured the continuity and smooth implementation of the intervention. Additionally, there was steady engagement with the stakeholder committee, including the chairperson, as well as with a patient panel and healthcare providers from various locations. Another key facilitator was the PPP adaptability regarding the programme’s implementation strategy in response to emerging needs. Also, a facilitator was conducting regular check-ins with external sites. This strategy was key for gathering information and maintaining proactive communication. Participants highlighted the critical role of being engaged and consistently checking in to identify and address potential issues early on, ensuring that the process did not stagnate.

The implementation faced several barriers, mainly due to the establishment of a newly formed PPP. A key barrier was the substantial effort needed to lay down the PPP’s operational structure and ensure that all team members were informed and committed to the agreed procedures. Participants explained that transparent communication and dialogue were essential to overcoming political complexities and aligning goals, as it reduced common PPP frustrations, enhancing the PPP’s impact. Additionally, another key barrier was unclear roles and responsibilities amongst PPP team members, especially regarding implementation tasks, which led to confusion and a lack of clarity on who was accountable for specific tasks. Finally, a key barrier was the limited PPP staffing capacity, which hindered effective programme implementation. This shortage in staffing capacity resulted in a lack of continuity and consistency throughout the process. Participants highlighted the necessity of having a dedicated and well-trained team, with adequate time allocated for implementation activities to ensure the successful implementation of the BENEFIT programme. The dynamic nature of the external environment and internal staff changes (i.e. illness and turnover) were also explained as contributing factors to the challenges, emphasising the need for a stable and well-supported team to navigate the complexities of the implementation process. However, another reason was the lack of a predetermined agreement on budget allocation for implementation between the public and private entities and the division of financial and personnel contributions.

## Discussion

This study evaluated the implementation of the BENEFIT programme, a complex eHealth intervention developed by a PPP. Barriers and facilitators encountered during its implementation within routine cardiac care were explored. Key facilitators identified included programme adaptability, communication and planning within local teams, acute need for digital healthcare, dedicated PPP leadership, a diligent PPP meeting structure and the PPP ability to quickly change the implementation strategy. By leveraging these facilitators, sustainable implementation of complex eHealth interventions by PPPs can be realised, supporting better chronic disease management and improved quality of life. Key barriers identified, based on their significant impact on the implementation process and the times they were mentioned, were specific PPP challenges (i.e. frequently changing roles, unclear roles and responsibilities and limited staffing), workplace disruptions, poor IT integration and different programme implementation goals amongst CR sites.

Based on the identified key barriers, we identified several areas for improvement. Regarding the key barrier ‘specific PPP challenges’, issues identified were frequent role transitions, unclear roles and responsibilities and limited staffing capacity. Also, maintaining open PPP communication regarding goals and interests was found to be a challenge, largely due to its inherently political nature. Such issues are well-known challenges within PPPs [28–30]. Several approaches are possible to anticipate these challenges. First, PPP efficiency lies in effective risk management before the start of a project [29], by identifying potential risks, performing a risk analysis and evaluation of the potential impact and executing risk mitigation. To mitigate the identified challenges, amongst others, transparent budgeting and staffing agreements are helpful, alongside a clear definition of roles and responsibilities to avoid role strain, impaired job satisfaction, emotional exhaustion and diminished organisational efficiency and commitment [31–33]. Second, participants’ insights, corroborated by the existing literature, highlight the importance of fostering an environment that prioritises clear, honest dialogue to navigate political complexities and align goals and motives of PPP parties. This approach builds trust and leads to consensus, higher efficiency and cost-effectiveness due to more effective and efficient collaboration [33]. Third, to enhance the PPP’s foundation, early engagement of expert advice [33], such as an organisational consultant, could be beneficial to provide strategic guidance on establishing efficient workflows, communication methods and collaboration techniques. Finally, additional strategies include promotion of autonomy [34], ensuring leadership commitment [35], encouraging collaborative team building and task mastery [36, 37] and regularly assessing and adapting team roles [37]. Implementing effective feedback systems, consistent education, training and access to information are also crucial strategies to move intervention deliverers from unengaged to fully committed users of the intervention [38].

Concerning the key barrier ‘workplace disruptions’, the COVID-19 pandemic significantly disrupted the participating healthcare organisations. This was compounded by the need to employ the BENEFIT programme differently than initially planned due to rapid tele-healthcare necessity. This extra time and energy investment led to workplace resistance, which hindered the implementation of the BENEFIT programme. The capacity of organisations to endure and thrive amidst chaotic conditions greatly depends on how well information system strategies are aligned with the organisation’s core operations [39]. To realise effective adaptation to changing circumstances, compatibility of all systems with current clinical workflows and technical infrastructures is crucial; seamless integration between different information system systems and infrastructures is necessary for smooth data flow and user experience. Additionally, organisations stay flexible, adaptive and well-prepared for changing circumstances by encouraging the use of different systems or programmes, such as complex eHealth interventions, as an integral part of healthcare delivery [40] and fostering a culture of innovation where staff can propose and test new ideas [41]. Concerning workplace resistance, excessive recent changes within organisations can result in an overwhelming sense of yet another implementation, also known as ‘initiative fatigue’ [42]. Despite understandably limited capacity, higher-ups had to make difficult choices, which led to top-down decision-making. Even though top-down decision-making is prevalent, bottom-up communication is essential to gather employee feedback during the initial stages of change processes and empower employees to contribute to the change process [43]. Furthermore, a strong implementation climate is needed to realise implementation success, which can be reached by organisational endorsement, supportive behaviours, rewarding cooperation, timely training and knowledge dissemination [44]. In response to challenges such as COVID-19, supportive management strategies and organisational endorsement could include providing mental health support services adapted to the needs of healthcare professionals, such as weekly group sessions with a counsellor [45], providing targeted, timely training sessions tailored to the new telehealth requirements to help staff feel prepared and confident in adapting to the sudden changes [46, 47], establishing rapid, transparent communication channels to keep all employees informed and engaged and mitigate potential uncertainties and ambiguities [48] and reallocating resources to areas of urgent need, e.g. by using an ethical decision-making tool [49].

Regarding the key barrier ‘poor IT integration’, integrating the BENEFIT programme into existing work structures was challenging due to poor programme integration within local IT systems. When implementing new eHealth initiatives, there is often a discrepancy between the eHealth innovation and the technological infrastructures present within healthcare organisations. eHealth adoption depends on the compatibility with current workflows and technical infrastructures [50–55]. Workflow-related issues play a major role in adopting eHealth innovations, as does effort expectation (e.g. ease of use). When eHealth innovations fit well within current workflows and infrastructure, they are easier to use and, thereby, more effective. Studies also show that incompatibility can lead to improvised solutions, reduced operational efficiency and potential adverse effects on patient care [54, 55]. To facilitate eHealth technology integration, healthcare organisations can adapt their operational protocols by applying strategic change management to address the multifaceted nature of healthcare delivery and the varied spectrum of professional roles involved [56]. Moreover, alignment of IT infrastructures should be a key focus in the early stages of designing eHealth innovations to ensure interoperability of systems. Using an iterative process involving end user feedback significantly improves usability and adoption of eHealth innovations [57].

Finally, concerning the key barrier ‘different programme implementation goals amongst CR sites’, we found that the primary reason for implementing the BENEFIT programme within organisations was found to be a source of confusion amongst participants, limiting engagement and involvement of healthcare providers in the process. This ambiguity may have resulted from the programme's complexity and continuous development during the research project. Whilst the development allowed customisation to local needs, it introduced elements of unpredictability and confusion. Interventions, especially within healthcare, can be inherently complex due to their multiple features, parties involved and varying contexts in which they are implemented [1]. This multiplicity can make the intervention ambiguous. Nevertheless, the complexity of such programmes can foster commitment when they are seen as fundamental changes rather than mere add-ons [37]. To address ambiguity, strategies such as developing a communication strategy that clearly defines and restricts the programme’s vision and use and ensures a shared understanding amongst all parties involved are essential [58], together with early involvement of key stakeholders, e.g. using participatory approaches to build consensus, to help define the primary way of using the intervention within the organisation [59]. Furthermore, establishing a transparent documentation process that records the evolution of programme vision and use is vital. This ensures that all changes are communicated promptly and clearly, aligning stakeholder perceptions and maintaining the programme’s relevance and effectiveness in practice.

### Strengths and limitations

A strength of our study lies in conducting semistructured interviews to collect rich, context-specific dynamics within the PPP and the BENEFIT programme implementation. Additionally, using the CFIR in our analytical approach was central to the systematic identification and categorisation of influencers across multiple levels of the BENEFIT programme implementation. Moreover, this study represents one of the first instances where CFIR has been employed to evaluate a PPP, paving the way for its broader application in similar research contexts. The multilayered nature of PPPs, with their complex interplay of public and private sector dynamics, makes CFIR particularly suitable for evaluation in such settings.

A limitation of our study is a potential recall bias amongst participants since the interviews occurred after the BENEFIT programme implementation had just ended, a process which took 2 years. Evaluation at the end could have influenced the accuracy of participants’ recollections regarding the implementation activities at the start of the process. This also meant that some potentially interesting participants were no longer available to interview because they had been employed elsewhere. Additionally, the focus on CFIR constructs through template analysis, apart from three added constructs, might have constrained the identification of new themes beyond the CFIR framework. This approach could have omitted factors not covered by CFIR, which may have influenced the implementation process. However, we tried to mitigate this by adding extra constructs during coding. Finally, our study featured a relatively small sample size. Nevertheless, we captured a diverse array of perspectives by involving all key stakeholders, amongst others, many participants with dual roles within the PPP, ensuring a broad representation of viewpoints. This choice, however, might have limited the generalisability of the results, as these individuals were deeply involved in the implementation process. They may have had different perspectives from stakeholders who were, e.g. within the consortium but not part of the implementation process, or end users such as individuals with CVD. Nevertheless, because we aimed to conduct a thorough evaluation of the implementation process, we intentionally selected participants who were actively engaged in the implementation process, ensuring rich and detailed insights into the challenges and facilitators experienced firsthand.

### Future perspectives

After realising the successful implementation of the BENEFIT programme or similar complex eHealth interventions, ensuring long-term sustainability is critical for continued success and impact. Maintenance strategies must be embedded in routine clinical practice to support lasting usage. This can include continuous training and education for healthcare providers to keep them engaged and proficient in using the intervention [44]. Integrating the eHealth intervention into existing clinical workflows and IT systems should also be revisited periodically to ensure compatibility and ease of use, as seamless integration reduces friction and enhances the likelihood of sustained use [52, 55]. Moreover, ongoing stakeholder engagement, including input from both end users and healthcare providers, is crucial for adapting the intervention to evolving needs and preferences [33]. Collecting regular user feedback through surveys or focus groups can guide iterative improvements [38, 57]. Finally, fostering a culture of innovation and support within healthcare organisations, where staff are encouraged and empowered to contribute ideas for enhancement, can further bolster the intervention’s maintenance within practice [41]. By implementing such strategies, the BENEFIT programme and comparable initiatives can achieve lasting impact, supporting chronic disease management and improving patient outcomes over time.

## Conclusion

This case study systematically examined facilitators and barriers encountered during the implementation of the BENEFIT programme, a complex eHealth intervention within routine cardiac care developed and implemented by a PPP. Our findings underscore the complexity of deploying such interventions within healthcare systems and offer critical insights for future endeavours. Key facilitators identified include the programme’s inherent adaptability, communication and planning within local teams, acute demand for digital healthcare, dedicated PPP leadership, a diligent PPP meeting structure and the PPP’s implementation strategy flexibility. These elements were instrumental in navigating implementation challenges and enhancing the programme’s acceptance and effectiveness. The study also identified key barriers that posed challenges to implementation, with the newly formed nature of the PPP mainly introducing specific challenges. Amongst others, frequently changing roles, unclear roles and responsibilities and limited staffing capacity were found to be PPP challenges. Additionally, workplace disruptions, poor IT integration and ambiguous implementation goals amongst different CR sites were found key barriers. Our findings provide valuable insights for future implementations of complex eHealth interventions, developed by PPP. The need for a comprehensive approach that accounts for the specific dynamics of PPPs appears to be essential, which includes the benefits of combined expertise and resources alongside the necessity for importance of clear role definition and resource allocation and the need for flexible, adaptive strategies to navigate diverse political landscapes within PPPs. This way, healthcare organisations can gain a deeper understanding of the specific dynamics involved in PPPs and use these insights to optimise the implementation of complex eHealth interventions into clinical practice. By addressing both barriers and facilitators systematically, future efforts can be better informed, enhancing the potential for successful integration and long-term sustainability of these interventions.

## Data Availability

The data used and analysed during the current study are available from the corresponding author upon reasonable request.

## Appendix 1

**Interview questions implementation evaluation BENEFIT programme**

**A: General and implementation-assigned roles, responsibilities and tasks**

1. What position do you have within your organisation?
2. Can you briefly tell what tasks are involved?
3. What is or was your task when it comes to the implementation of the BENEFIT programme?
4. Did you have tasks within the implementation process that fell outside your regular role as a health care provider/employee?
5. Did you have any other tasks in the implementation process?
6. From an organisational perspective, what is or was your primary goal regarding the use of the BENEFIT programme?
7. When would implementing the BENEFIT programme be successful; what conditions are needed?

**B: BENEFIT programme implementation, questioning barriers and facilitators**

Innovation domain (at the time: Intervention characteristics)

1. What have been the most significant successes regarding the innovation?
2. What are the biggest challenges you encountered regarding the innovation?
3. Regarding your role:
  - Can you indicate what succeeded thanks to your contribution?
  - Can you provide some reason(s) for this?
  - And what went less well and why?

Outer setting domain

1. What have been the most significant successes regarding the outer setting?
2. What are the biggest challenges you encountered regarding the outer setting?
3. Regarding your role:
  - Can you indicate what succeeded thanks to your contribution?
  - Can you provide some reason(s) for this?
  - And what went less well and why?

Inner setting domain

1. What have been the most significant successes regarding the inner setting?
2. What are the biggest challenges you encountered regarding the inner setting?
3. Regarding your role:
  - Can you indicate what succeeded thanks to your contribution?
  - Can you provide some reason(s) for this?
  - And what went less well and why?

Individuals’ domain (at the time: Characteristics of individuals)

1. What have been the most significant successes for you personally? And for your team?
2. What are the biggest challenges you encountered personally? And for your team?
3. Regarding your role:
  - Can you indicate what succeeded thanks to your contribution?
  - Can you provide some reason(s) for this?
  - And what went less well and why?

Implementation process domain (at the time: Process)

1. What have been the most significant successes regarding the implementation process?
2. What are the biggest challenges you encountered regarding the implementation process?
3. Regarding your role:
  - Can you indicate what succeeded thanks to your contribution?
  - Can you provide some reason(s) for this?
  - And what went less well and why?

## Appendix 2

**Coding tree based on the CFIR constructs**

## Appendix 3

**Consolidated criteria for reporting qualitative studies: 32-item checklist**

**Table:**

Item number	Guide questions/description	Page
**Domain 1: Research team and reflexivity**		
Personal Characteristics		
1. Interviewer/facilitator	Which authors conducted the interview or focus group?	p. 3
2. Credentials	What were the researcher’s credentials? For example, PhD, MD	MSc; p. 1
3. Occupation	What was their occupation at the time of the study?	p. 3
4. Gender	Was the researcher male or female?	p. 3
5. Experience and training	What experience or training did the researcher have?	p. 3
Relationship with participants		
6. Relationship established	Was a relationship established prior to study commencement?	p. 3
7. Participant knowledge of the interviewer	What did the participants know about the researcher? For example, personal goals, reasons for doing the research	p. 3
8. Interviewer characteristics	What characteristics were reported about the interviewer/facilitator? For example, Bias, assumptions, reasons and interests in the research topic	p. 3
Domain 2: Study design		
Theoretical framework		
9. Methodological orientation and theory	What methodological orientation was stated to underpin the study? For example, grounded theory, discourse analysis, ethnography, phenomenology, content analysis	p. 3
Participant selection		
10. Sampling	How were participants selected? For example, purposive, convenience, consecutive, snowball	p. 3
11. Method of approach	How were participants approached? For example, face-to-face, telephone, mail, e-mail	p. 3
12. Sample size	How many participants were in the study?	p. 3
13. On-participation	How many people refused to participate or dropped out? Reasons?	p. 3
Setting		
14. Setting of data collection	Where was the data collected? For example, home, clinic, workplace	p. 3
15. Presence of non-participants	Was anyone else present besides the participants and researchers?	p. 3
16. Description of sample	What are the important characteristics of the sample? For example, demographic data, date	p. 3,4
Data collection		
17. Interview guide	Were questions, prompts, guides provided by the authors? Was it pilot tested?	Appendix 1
18. Repeat interviews	Were repeat interviews carried out? If yes, how many?	p. 3
19. Audio/visual recording	Did the research use audio or visual recording to collect the data?	p. 3
20. Field notes	Were field notes made during and/or after the interview or focus group?	p. 3
21. Duration	What was the duration of the interviews or focus group?	p. 3
22. Data saturation	What was the duration of the interviews or focus group?	p. 3
23. Transcripts returned	Were transcripts returned to participants for comment and/or correction?	p. 3
Domain 3: Analysis and findings		
Data analysis		
24. Number of data coders	How many data coders coded the data?	p. 4
25. Description of the coding tree	Did authors provide a description of the coding tree?	p.4 and Appendix 2
26. Derivation of themes	Were themes identified in advance or derived from the data?	p. 4
27. Software	What software, if applicable, was used to manage the data?	p. 4
28. Participant checking	Did participants provide feedback on the findings?	p. 4
Reporting		
29. Quotations presented	Were participant quotations presented to illustrate the themes/findings?	pp. 4-7+ Tables DI–DV and Appendix 4
30. Data and findings consistent	Was there consistency between the data presented and the findings?	pp. 4–7 + Tables DI–DV
31. Clarity of major themes	Were major themes clearly presented in the findings?	pp. 4–7 + Tables DI–DV
32. Clarity of minor themes	Is there a description of diverse cases or discussion of minor themes?	pp.4–7 and Tables DI–DV and Appendix 4

## Appendix 4

**Other factors influencing implementation**

**CFIR Domain 1: Innovation**

**Table DI.** Factors of CFIR Domain 1: Innovation influencing implementation

Theme	Quotes
**Facilitators**	
Design and source of the programme	‘The programme provides a holistic approach to lifestyle modification, so depending on what someone needs, you can offer something. Because of the PPP, we have been able to connect so many partners in the ecosystem that you can refer patients to within the intervention. This enhances its appeal. Besides that, the owners also developed all kinds of things, such as the challenges in the portal.’ ‘Due to the PPP, the BENEFIT programme is co-created with many healthcare providers, academic researchers, and business developers. We’ve engaged in extensive dialogue, gathering feedback regarding needs and desires, and then cross-referenced this input with literature to merge the necessary elements effectively.’
Sustainable business model	‘The BENEFIT intervention, as developed now, does not incur losses for hospitals. It is feasible to offer the programme cost-effectively, for example through a third party, enabling hospitals to economise and subcontract the service provision.’
**Barriers**	
Programme complexity	‘The programme’s complexity lies in its multi-layered connections. We noticed that patients didn’t understand the goal so quickly, so I thought the programme was highly complex. Of course, the main goal is to empower patients, to get them moving, feel supported, and be able to find resources to continue working with their behavioural change, but that got overshadowed by the complexity.’
Digital skills of some patients	‘Surely, we sometimes overlook that many people still face challenges with online surveys. This varies by location, and it’s evident, isn’t it? In some locations, surveys are always completed, whilst in others, they are hardly ever.’

**Table DII.** Factors within CFIR Domain 2: Outer setting influencing implementation

Theme	Quotes
**Facilitator**	
Focus on prevention in policies and law	‘The government has intensified its focus on preventative measures. Whilst this didn’t necessarily translate to aligned reimbursements, prevention has, for the first time, been prioritised on the agenda and is now uniquely covered by basic insurance. This advancement coincided with the BENEFIT programme implementation.’
**Barrier**	
Commitment to external deadlines	‘There are immutable deadlines, and one must deliver eventually, ready or not. Despite suboptimal conditions or an imperfect product, action must be taken, otherwise, the project concludes, time expires, and nothing is accomplished.’

**Table DIII.** Factors within CFIR Domain 3: Inner setting influencing implementation

Theme	Quotes
**Facilitator**	
Mission alignment	‘A distinct advantage for us was the pre-existence of the programme, obviating the need for its creation from scratch, which was a considerable blessing.’ ‘The idea of extending the three-month cardiac rehabilitation trajectory with an ongoing coaching element was immensely appealing to us, and we embraced it with enthusiasm.’
**Barrier**	
Integration of the programme into existing workflows and practice	‘The programme supplements existing healthcare services, adding to an already demanding schedule. It required substantial energy and time, particularly as we navigated a suitable and effective working methodology within our organisation. And encountering and resolving unforeseen challenges was an energy-intensive process.’

**Table DIV.** Factors of CFIR Domain 4: Individuals influencing implementation

Theme	Quotes
**Facilitators**	
Dedicated employees	‘The programme’s good intentions were apparent to all serving as a strong motivator. This encouraged a willingness to invest and participate, an effort we executed commendably, I believe.’
Confidence in using the programme	‘There was a consensus on the programme’s utility. However, as it became apparent that further refinement was needed, there was a palpable decline in enthusiasm amongst nurses and physiotherapists, who faced repeated hindrances due to updates. Our collaborative efforts did enhance the platform, but each improvement phase came with a cost to satisfaction levels, impacting the healthcare provider’s eagerness to utilise it.’
Having local implementation project leaders	‘My presence here is to ascertain which tasks need execution and who will undertake them, whilst also establishing the necessary preconditions. We have maintained extensive discussions with [L10] concerning the technical set-up, allowing healthcare providers to concentrate on the clinical aspects. My role is pivotal in ensuring that required actions are methodically executed, not all at once, but in a sequenced manner: identifying who is responsible for each task.’ ‘In collaboration with [P2], we gave presentations. We proposed that [P2] joined to offer insights from the research perspective, which was essential and beneficial. They were receptive to this idea, and our interactions were invariably productive. This synergy was crucial, as our mutual dependence was evident.’
**Barriers**	
Workplace resistance	‘When obstacles arise, my role is to guide decision-making: determining what will be done and what won’t. At times, this involves asserting that certain actions are non-negotiable, overcoming resistance because these steps are necessary.’
Top-down decision-making	‘Decisions made by the board are definitive, and their commitment to resolving issues and progressing with the initiative was evident. However, this sometimes conflicted with personal confidence and motivation. Nonetheless, the decision was to forge ahead.’
Employee compliance over conviction	‘Having not been part of the organisation for long, I tend to align with the existing expertise within the team. They opt for a particular course of action, believing it will achieve the desired outcomes, despite my colleague and I expressing reservations. Ultimately, one defers to the judgment and experience of those in senior positions.’

**Table DV.** Factors of CFIR Domain 5: Implementation process influencing implementation

Theme	Quotes
**Facilitators**	
Regular check-ins with external sites	‘Engaging with individuals and providing clear instructions is crucial. Equally important is the ongoing dialogue to check in on progress, identify challenges, and ensure proactive problem-solving. Experience has taught us that processes invariably encounter stagnation; anticipating where and why, however, remains unpredictable.’
Face-to-face introduction sessions	‘Initiating interactions with face-to-face introduction sessions is immensely beneficial. It establishes an understanding of the individuals you are working with, their motivations, and interests, which is fundamental to building mutual trust.’

Table DI describes all factors that proved to be relevant during the implementation, along with exemplary quotes from participants illustrating these factors. A facilitator was the comprehensive design of the programme and its development via cocreation with academia, healthcare providers, experts and IT developers. The programme encompassed various lifestyle interventions and a rewards programme to incentivise health behaviours and adherence to make healthy living. These features were possible due to the availability of an extensive network of partners within the PPP, which stemmed mainly from the private company’s considerable pre-existing network. Finally, another facilitator was the development of a sustainable business model which ensured that hospitals could offer the programme without incurring financial losses, further facilitating large-scale programme implementation. Development of this business model was possible due to the private party focusing on finding a sustainable financing structure and the expansive knowledge, expertise and network of the PPP. However, programme complexity was a barrier, particularly regarding patient understanding of the PHA. According to several participants, the multilayered connections and options within the PHA made it difficult for some patients to understand the main functions and use of the PHA, potentially hindering their ability to fully engage with and benefit from the programme. Finally, another barrier was the digital skills of a minority of patients included in the programme. Patients’ digital skills varied widely, with noticeable disparities between locations. Since all surveys for programme participation could only be completed digitally, this entailed additional support from healthcare providers and administrative staff.

**CFIR Domain 2: Outer setting**

Table DII presents the identified factors, along with exemplary quotes. A facilitator was the government’s increasing focus on preventive measures, which became visible through significant policy changes. At the end of the BENEFIT implementation, preventive healthcare was included in healthcare insurance coverage, representing a significant advancement in officially recognising the importance of preventive healthcare. However, a barrier identified was the commitment to external deadlines, as the implementation had to proceed despite not being in an ideal state due to COVID-19. This led to higher-ups experiencing tension between project timelines and readiness of the intervention.

**CFIR Domain 3: Inner setting**

Table DIII describes all identified factors, along with exemplary quotes. Mission alignment was a facilitator; participants were enthusiastic about the programme already existing, so organisations did not need to create a new initiative, and the extension of a 3-month CR programme by a coaching element was met with enthusiasm, as participants believed in the potential benefits of this approach. However, a key barrier was poor IT integration; participants reported significant challenges due to duplicate filing and the need to work across multiple systems, which led to increased workload and inefficiencies. Additionally, assimilating the programme into existing workflows was a barrier. The additional workload required to integrate the BENEFIT programme into existing workflows was highlighted as a considerable barrier, requiring substantial time and energy investment, particularly in the context of already busy work schedules.

**CFIR Domain 4: Individuals**

Table DIV describes all identified factors, along with exemplary quotes. A facilitator in specific organisations was strong dedication amongst employees, creating a supportive environment for its implementation. Employees across various roles recognized the positive intentions behind the programme, which motivated them to invest their time and effort into making the programme implementation a success. This collective dedication also played a significant role in navigating the initial stages of the implementation, setting a positive tone for the continuation of the process. Furthermore, within these organisations, a facilitator was the presence of confidence amongst employees in using the BENEFIT programme. This confidence, however, was not without its challenges, as it was tested during periods of adapting and updating the PHA. Nevertheless, commitment was evident, and it played a crucial role in maintaining a level of enthusiasm and willingness to participate in the process. Finally, a facilitator was local implementation project leaders who provided clear direction and coordination for the necessary tasks and activities. This role was crucial in ensuring that all preconditions were met and healthcare providers could focus on patient care whilst logistical and administrative aspects were handled effectively. These leaders were willing to go beyond their primary responsibilities, amongst others, by sharing their work time between different roles and responsibilities. This was crucial for overcoming (unforeseen) challenges and continuing implementation.

A barrier identified was workplace resistance. Some higher-ups felt that they had to impose tasks on employees to keep the implementation on track. This involved making tough decisions about what to prioritize and set aside. Furthermore, in some organisations, a key barrier to implementation was top-down decision-making. Whilst there was commitment from higher-ups to sort issues out and progress with the implementation, a top-down approach led to a lack of confidence and motivation amongst employees. Being obligated to comply without much input affected overall morale and enthusiasm towards the implementation. Finally, a barrier was employee compliance over conviction. Especially amongst newer employees, e.g. within the role of implementation facilitator, there existed a tendency to go along with the suggested deadlines or implementation strategies of higher-ups despite having different personal views of what was necessary and possible within the given timeframe. Newer employees relied largely on existing in-house experience and knowledge, leading to a dynamic where suggested approaches and strategies by higher-ups or more experienced team members were not fully aligned with their beliefs, but nevertheless adopted without genuine engagement or confidence in their efficacy. It sometimes led to their premature communication of deadlines to external stakeholders, based on upper management’s directives, despite internal expectations that they were unrealistic and likely to be missed.

**CFIR Domain 5: Implementation process**

Table DV describes all identified facilitators, along with exemplary quotes. A facilitator was conducting regular check-ins with external sites. This strategy was key for gathering information and maintaining proactive communication. Participants highlighted the critical role of being engaged and consistently checking in to identify and address potential issues early on, ensuring that the process did not stagnate. Additionally, initiating the implementation with face-to-face introduction sessions was a facilitator, creating a foundation of trust and mutual understanding amongst team members, which facilitated smoother interactions and cooperation throughout the process.
